# Ovariectomy Induces a Shift in Fuel Availability and Metabolism in the Hippocampus of the Female Transgenic Model of Familial Alzheimer's

**DOI:** 10.1371/journal.pone.0059825

**Published:** 2013-03-26

**Authors:** Fan Ding, Jia Yao, Liqin Zhao, Zisu Mao, Shuhua Chen, Roberta Diaz Brinton

**Affiliations:** 1 Department of Pharmacology and Pharmaceutical Sciences, School of Pharmacy, University of Southern California, Los Angeles, California, United States of America; 2 Department of Neurology, Keck School of Medicine, University of Southern California, Los Angeles, California, United States of America; University of Valencia, Spain

## Abstract

Previously, we demonstrated that reproductive senescence in female triple transgenic Alzheimer's (3×TgAD) mice was paralleled by a shift towards a ketogenic profile with a concomitant decline in mitochondrial activity in brain, suggesting a potential association between ovarian hormone loss and alteration in the bioenergetic profile of the brain. In the present study, we investigated the impact of ovariectomy and 17β-estradiol replacement on brain energy substrate availability and metabolism in a mouse model of familial Alzheimer's (3×TgAD). Results of these analyses indicated that ovarian hormones deprivation by ovariectomy (OVX) induced a significant decrease in brain glucose uptake indicated by decline in 2-[^18^F]fluoro-2-deoxy-D-glucose uptake measured by microPET-imaging. Mechanistically, OVX induced a significant decline in blood-brain-barrier specific glucose transporter expression, hexokinase expression and activity. The decline in glucose availability was accompanied by a significant rise in glial LDH5 expression and LDH5/LDH1 ratio indicative of lactate generation and utilization. In parallel, a significant rise in ketone body concentration in serum occurred which was coupled to an increase in neuronal MCT2 expression and 3-oxoacid-CoA transferase (SCOT) required for conversion of ketone bodies to acetyl-CoA. In addition, OVX-induced decline in glucose metabolism was paralleled by a significant increase in Aβ oligomer levels. 17β-estradiol preserved brain glucose-driven metabolic capacity and partially prevented the OVX-induced shift in bioenergetic substrate as evidenced by glucose uptake, glucose transporter expression and gene expression associated with aerobic glycolysis. 17β-estradiol also partially prevented the OVX-induced increase in Aβ oligomer levels. Collectively, these data indicate that ovarian hormone loss in a preclinical model of Alzheimer's was paralleled by a shift towards the metabolic pathway required for metabolism of alternative fuels in brain with a concomitant decline in brain glucose transport and metabolism. These findings also indicate that estrogen plays a critical role in sustaining brain bioenergetic capacity through preservation of glucose metabolism.

## Introduction

Previously we demonstrated that a decline in mitochondrial bioenergetics precedes the development of AD pathology in the female triple transgenic Alzheimer's (3×TgAD) mouse model [1]. In both normal and 3×TgAD mice, reproductive senescence, both natural and ovariectomy-induced, resulted in a significant decline in aerobic glycolysis, PDH, and Complex IV cytochrome c oxidase activity, and mitochondrial respiration. Following transition through reproductive senescence, enzymes required for long-chain fatty acid (HADHA) and ketone body (SCOT) metabolism were significantly increased whereas cytochrome c oxidase (Complex IV) collapsed by 40% in both the nonTg and 3×TgAD brain which was predictive of a concomitant decline in ATP generation. These bioenergetic changes, observed during natural reproductive senescence, were recapitulated in an ovariectomy model of menopause [2].

Consistent with findings from basic science discoveries, data emerging from clinical positron emission tomography with 2-[^18^F]fluoro-2-deoxy-D-glucose (FDG-PET) analyses indicate a progressive reduction in cerebral glucose metabolic rate (CMRglu), particularly in posterior cingulate (PCC) and parietal-temporal cortex, in persons with Alzheimer's disease as well as those at increased risk for AD [3]–[7]. Clinical imaging has also indicated a spatial correlation between increased aerobic glycolysis and β-amyloid deposition in the “default mode network” brain areas, suggesting that deficits in energy supply may underlie the vulnerability to the AD pathogenic process in such areas [8], [9]. Further in persons with AD, compromised brain glucose metabolism is accompanied by parallel activation of alternative metabolic pathways, as evidenced by a utilization ratio of 2∶1 glucose to alternative substrate in persons with incipient AD compared to a ratio of 29∶1 in healthy elderly controls [10].

Earlier studies indicated that 17β-estradiol (E2) promoted glucose uptake [11], glycolysis [12], glycolytic-coupled tricarboxylic acid cycle (TCA) function, mitochondrial respiration and ATP generation [2], [13]–[15]. Results of these discovery analyses demonstrate that 17β-estradiol sustains the ability of the brain to transport and utilize glucose as its primary fuel source. From a translational perspective, these basic science findings are supported by clinical analyses of glucose metabolism in menopausal women. Postmenopausal women on estrogen therapy were reported to have increased cerebral blood flow and cerebral metabolism relative to non-users [2], [16]–[18]. Further, non-users exhibited a significant decline in glucose metabolic rate, particularly in the posterior cingulate and prefrontal cortex, which closely resembled the hypometabolic profile of AD brains [17], [18]. Collectively, both preclinical analyses in animal models [1], [2], [19] and clinical observations [5]–[7], [17] provide compelling evidence in support of decline in bioenergetic function in brain as an early indicator of neurodegenerative risk.

In the present study, we sought to investigate the impact of loss of ovarian hormones and the efficacy of 17β-estradiol to sustain aerobic glycolysis as a primary bioenergetic system of the brain. Specifically, we determined whether these bioenergetic deficits were in response to a decline in substrate availability as evidenced by capacity for glucose uptake, substrate (glucose, lactate/ketone body) transporter expression and/or enzyme systems required for glucose metabolism. The goal of these analyses was to determine the initiating events that lead to a dysfunctional bioenergetic system in brain. As a translational preclinical model relevant to reproductive aging in the human, we utilized temperature and body weight dysregulation in the ovariectomized 3×TgAD mouse model as the trigger to initiate analyses of brain metabolism. Results presented herein demonstrate that in 3×TgAD mice, ovarian hormone loss caused by OVX induces a decline in brain glucose uptake, which could be partially attributed to decreased glucose transporter expression and compromised hexokinase activity. The OVX induced decline in cerebral glucose metabolism was accompanied by the activation of alternative metabolic pathways. Further, E2 treatment initiated at the time of OVX prevented the bioenergetic fuel shift by sustaining key elements in glucose metabolism in brain.

## Materials and Methods

### Animal Treatments and Ethics

All rodent experiments were performed following National Institutes of Health guidelines on use of laboratory animals and an approved protocol (protocol number: 10217) by the University of Southern California Institutional Animal Care and Use Committee. The presented study has been approved by the University of Southern California Institutional Animal Care and Use Committee (Ethics Committee).

### Transgenic mice

Colonies of the 3×TgAD mice strain (129S; Gift from Dr. Frank Laferla, University of California, Irvine) [20] were bred and maintained at the University of Southern California (Los Angeles, CA) following National Institutes of Health guidelines on use of laboratory animals and an approved protocol by the University of Southern California Institutional Animal Care and Use Committee. Mice were housed on 12 hours light/dark cycles and provided ad libitum access to food and water. The characterization of amyloid and tau pathologies, as well as synaptic dysfunction in this line of mice has been described previously [20] and confirmed in our laboratory. Mice were genotyped routinely to confirm the purity of the colony. To ensure the stability of AD-like phenotype in the 3×TgAD mouse colony, we performed routine immunohistochemical assays every 3 to 4 generations. Only offspring from breeders that exhibit stable AD pathology were randomized into the study.

### Experimental design

**Figure 1:**
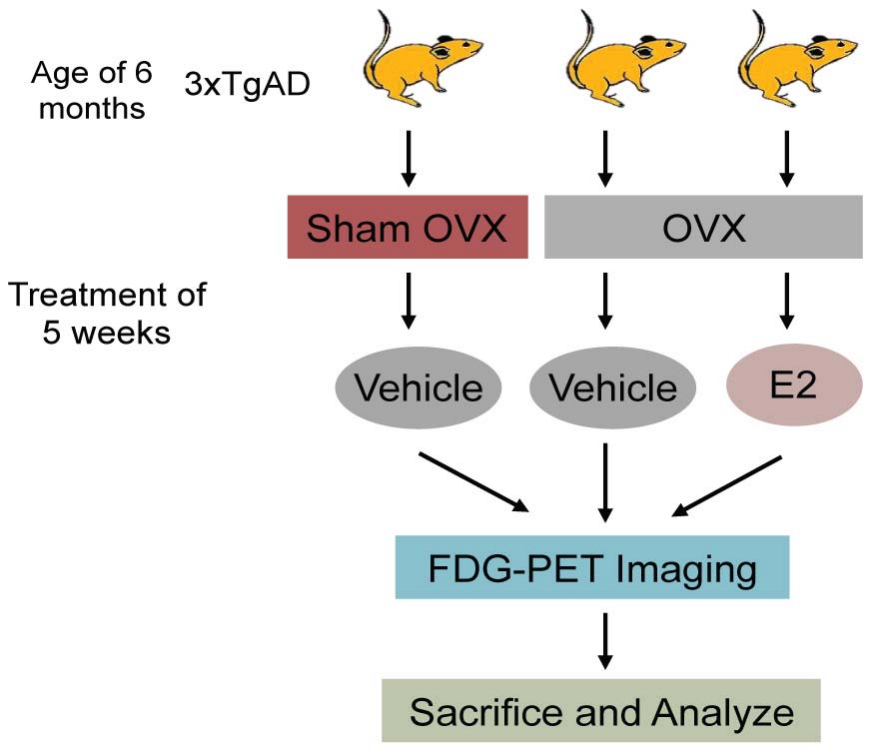
Experimental paradigm. 6-month-old 3×TgAD mice were randomly assigned into the following three treatment groups (n = 7 per group): sham ovariectomized (Sham-OVX), ovariectomized (OVX), and OVX plus 17β-estradiol (OVX+E2). Mice were bilaterally OVX and injected with corn oil or corn oil with E2 (50 µg/kg, Subcutaneously) daily for 5 weeks. Weight and tail skin temperature were monitored twice per week during the sleep cycle before FDG-microPET imaging. After FDG-microPET imaging, mice were sacrificed for *in vitro* analyses of substrate transport and utilization.

To investigate the change in substrate availability following removal of ovaries and treatment with 17β-estradiol(E2), 6-month-old 3×TgAD mice were randomly assigned into the following three treatment groups (*n* = 7 per group): sham ovariectomized (Sham-OVX), ovariectomized (OVX), and OVX plus 17β-estradiol (OVX+E2). Mice were bilaterally OVX and injected with corn oil or coin oil containing E2 (50 µg/kg, Subcutaneously) daily for 5 weeks (Fig.1).

### Body weight and tail skin temperature (TST) measurement

Body weight and tail skin temperature (TST) were monitored twice per week during the sleep cycle. To allow mice recover from the OVX surgery, measurements started one week after the surgery. TST measurements were conducted using an infrared thermometer (BIO-152-IRB, Bioseb, Chaville, France) designed for small rodents. TST was measured twice per week until an increase of 1 degree or higher of temperature was detected and sustained for 1 week. Body weight was also determined twice per week subsequent to skin tail temperature measurement. Following one week of sustained temperature dysregulation, mice underwent FDG-PET imaging.

### FDG-microPET and microCT imaging

Mice were maintained under anesthesia during microPET and microCT scans with 2-2.5% isoflurane in oxygen. Scans were performed in an imaging chamber equipped with a nose cone for anesthesia delivery and heating pad for body temperature control. MicroPET imaging was performed with a microPET R4 rodent model scanner (Concorde Microsystems Inc, Knoxville, TN) and micro CT imaging was performed on MicroCAT II tomography (Siemens Preclinical Solutions, Knoxville, TN). Mice were injected intravenously via the tail vein with radiotracer [^18^F] Fluoro-2-deoxy-2-D- glucose (FDG, 200 µCi, 100 uL). Radioactive dose was determined prior to injection by radioisotope dose calibrator (Capintec, CRC-712M). At 40 min post-injection of FDG, each mouse was positioned in the microPET scanner in the center of the 10.8 cm transaxial and 8 cm axial field of view (FOV). Brain microPET data were collected for 10 min followed by a 10 min microCT scan for the purpose of co-registration.

Co-registration of microPET and microCT data was performed using the AMIDE software package (http://amide.sourceforge.net/). After co-registration of the PET and CT images, ROI (region of interest) was defined and used to measure the radioactivity concentration in brains. Decay correction was used to adjust the actual radioactivity dosage injected (Actual radioactivity dosage at time of injection  =  Initial radioactivity ×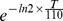, T = T minutes between injected time point and initial time point). Data were analyzed using Student's t-test with differences between groups with a p value<0.05 considered statistically significant.

### Brain tissue preparation and Western blot analysis

Upon completion of FDG-microPET imaging, 3×TgAD mice (*n* = 7 per group) were sacrificed and the brains rapidly dissected on ice. Hippocampus was processed for protein extraction using Tissue Protein Extraction Reagent (Thermo Scientific, Rockford, IL, USA) with phosphatase and protease inhibitors (Sigma, St. Louis, MO, USA), and protein concentrations were determined with the Bio-Rad Bradford assay. Equal amounts of protein (20 µg/well) were loaded in each well of a 12.5% SDS PAGE criterion gel (Bio-Rad, Hercules, CA) and electrophoresed with Tris/glycine running buffer. Proteins were transferred to 0.45 µm pore size polyvinylidene difluoride (PVDF) membranes and immuneblotted with GLUT1 (glucose transporter 1) antibody (1∶1500, Abcam, Cambridge, MA, USA), GLUT3 (glucose transporter 3) antibody (1∶1000, Abcam, Cambridge, MA, USA), Hexokinase II antibody (1∶1000, Millipore, Billerica, MD, USA), MCT2 (Monocarboxylate transporter 2) antibody (1∶1000, Millipore, Billerica, MD, USA), SCOT (3-oxoacid-CoA transferase) antibody (1∶100, Santa Cruz Biotechnology, Santa Cruz, CA, USA), LDHB (lactate dehydrogenase B) antibody (1∶1000, Abcam, Cambridge, MA, USA), and LDH V (lactate dehydrogenase V) antibody (1∶1000, Abcam, Cambridge, MA, USA). HRP-conjugated anti-rabbit antibody and HRP-conjugated anti-mouse antibody (Vector Laboratories, Burlingame, CA, USA) were used as secondary antibodies. Immunoreactive bands were visualized with Pierce SuperSignal Chemiluminescent Substrates (Thermo Scientific, Waltham, MA, USA) and captured by Molecular Imager ChemiDoc XRS System (Bio-Rad Laboratories, Hercules, CA, USA). All band intensities were quantified using the Un-Scan-it (version 6.0, Silk Scientific, Orem, UT, USA) software.

### Hexokinase activity assay

Hexokinase activity assay was measured by monitoring the conversion of NAD+ (nicotinamide adenine dinucleotide) to NADH (reduced nicotinamide adenine dinucleotide) by following the change in absorption at 340 nm. The assay medium contained: 0.1 µg/µL of the hippocampal tissue protein, 0.05 M Tris*HCl, PH 8.0, 13.3 mM MgCl2, 0.112 M glucose, 0.227 mM NAD+, 0.5 mM Adenosine 5′Triphosphate, and 1I U/mL glucose-6-phosphate dehydrogenase (*Leuconostoc mesenteroides*) in a final volume of 150 µL. The OD at λ = 340 nm was measured every 1 min for 30 mins at a temperature of 30°C. The increase in OD reflects the increase in NADPH concentration, and the total hexokinase activity was calculated from the slope of the resulting curve.

### Serum β-hydroxybutyrate measurement

The concentration of serum β-hydroxybutyrate was determined by β-hydroxybutyrate liquicolor assay kit (Stanbio laboratory, TX, USA) following manufacturer's instructions.

### Real-time RT-PCR gene expression profiling

Total RNA was isolated from mouse hippocampal tissues using the RNeasy kit (Qiagen, Valencia, CA). The quality and quantity of RNA samples were determined using the Experion RNA analysis kit (Bio-Rad, Hercules, CA). RNA samples were reverse-transcribed to cDNA using the high capacity cDNA reverse transcription kit (Applied Biosystems, Foster City, CA) and stored at −80°C for gene expression analysis. Taqman low-density arrays (TLDA) were custom manufactured at Applied Biosystems. Real-time RT-PCR experiment and data analysis were conducted following the methods as described in our previous report [21].

## Results

### Ovariectomy induced increase in body weight and tail skin temperature (TST): prevention by 17β-estradiol

It is well documented that ovarian hormone loss is associated with increased body weight and temperature dysregulation in menopausal women [22]. In this study, we used OVX mice as a translational model of surgically oophorectomized premenopausal women. In this model, body weight and tail skin temperature served as surrogate markers of metabolic and temperature dysregulation [23]

**Figure 2:**
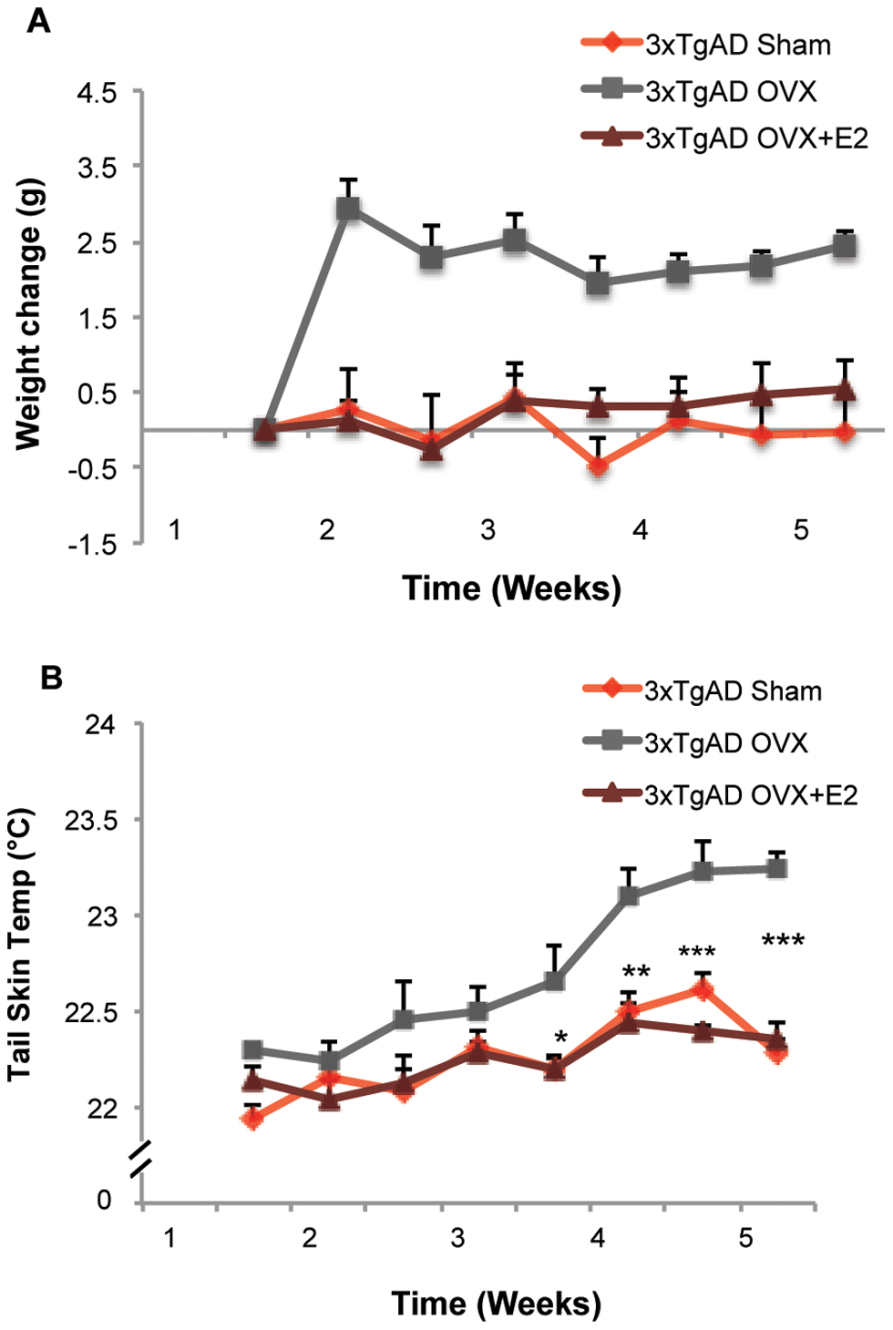
Ovariectomy (OVX) induced increase in body weight and tail skin temperature and prevention by 17β-estradiol. A. OVX induced a significant increase in body weight (0.27±0.54 vs. 2.94±0.39, F (2, 13) = 12.61, p<0.001, n = 5–6) one week after surgery, which was sustained through the duration of the experiment. E2 treatment (50 µg/kg, SC) initiated at time of OVX prevented the OVX-induced body weight gain. B. OVX induced a significant rise (22.28±0.07 vs. 23.24±0.08, F (2,13) = 42.21, p<0.001, n = 5–6) in tail skin temperature 4 weeks after surgery, and E2 treatment (50 µg/kg, SC) initiated at time of OVX prevented the OVX-induced increase in tail skin temperature.

OVX rapidly induced a significant increase in body weight (Fig. 2A; 2.94±0.39 (OVX group) vs. 0.27±0.54 (Sham-OVX group), F (2, 13) = 12.61, p<0.001, n = 5–6) one week after surgery and this increase was sustained throughout the duration of the experiment. E2 treatment initiated at the time of OVX prevented OVX-induced body weight increase. OVX also induced a significant rise in tail skin temperature 4 weeks after the OVX surgery, which reached asymptote by week 5. Asymptotic temperature triggered *in vivo* brain glucose imaging followed by *in vitro* analyses (Fig.1). Tail skin temperature of OVX animals increased 1 °C±0.03 °C (Fig. 2B; 23.24±0.08 (OVX group) vs. 22.28±0.07 (Sham-OVX group), F (2,13) = 42.21, p<0.001, n = 5–6) by week 5 relative to Sham-OVX animals. OVX-induced rise in tail skin temperature was prevented by E2 treatment initiated at the time of OVX.

### Ovariectomy induced decrease in brain glucose uptake: partial prevention by 17β-estradiol

**Figure 3:**
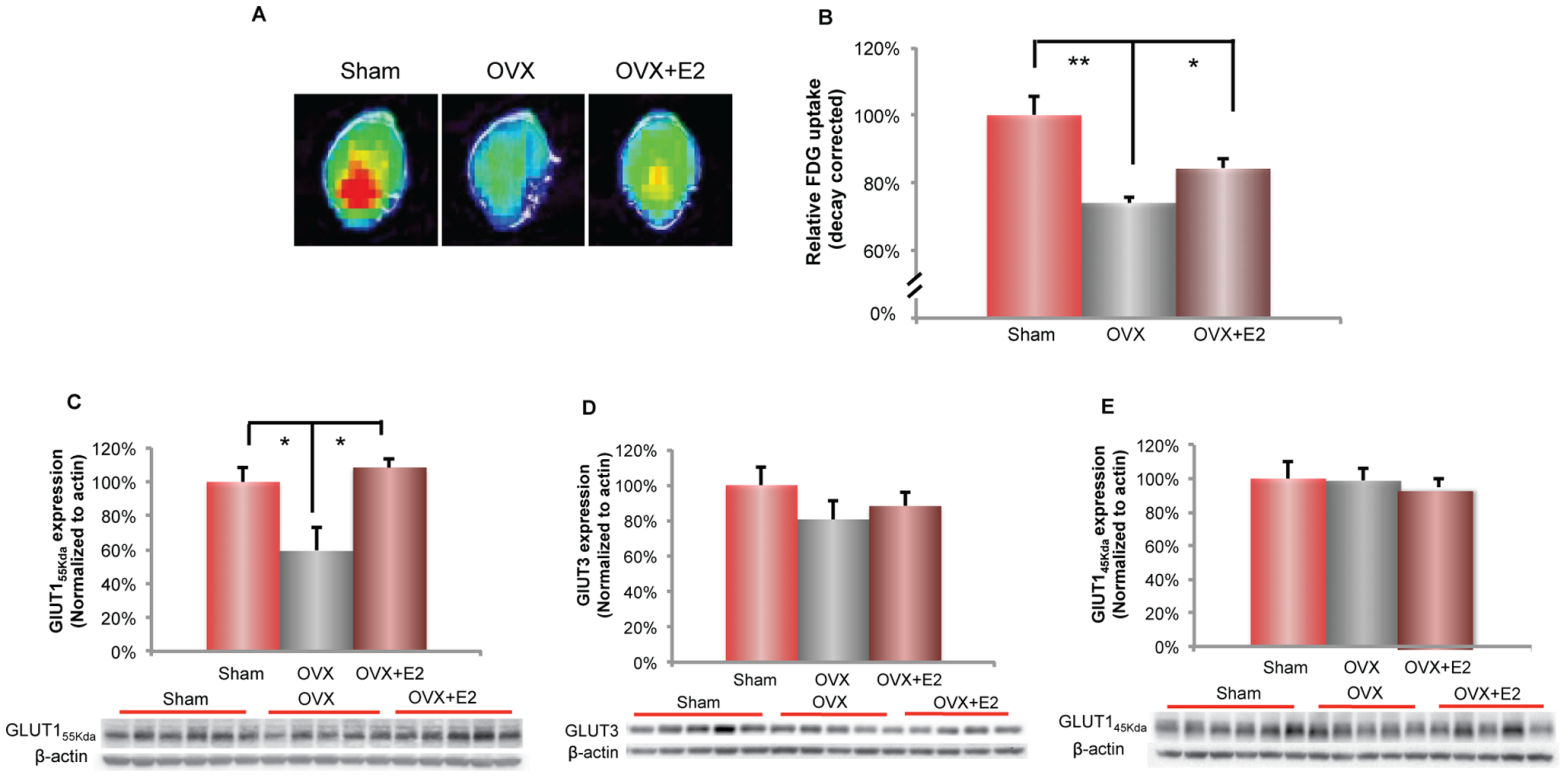
Ovariectomy (OVX) induced decline in brain glucose uptake and BBB GLUT1_55 Kda_ expression: prevention by 17β-estradiol. A. Representative FDG-microPET images showed a decline in brain glucose uptake in OVX condition, which was partially prevented by E2 treatment (50 µg/kg, SC). (Red and yellow indicate higher values; green and blue indicate lower values). B. Quantitative analysis of brain glucose uptake demonstrated a significant decrease in OVX condition (**, p<0.01, bars represent mean value±SEM, n = 5–7), which was partially prevented by E2 treatment (50 µg/kg, SC) (*, p<0.05; bars represent mean value±SEM, n = 5–6). C. OVX induced a significant decrease in protein expression of blood brain barrier glucose transporter 1 (GLUT1_55 Kda_), which was prevented by E2 treatment (50 µg/kg, SC). (*, p<0.05, bars represent mean value ± SEM, n = 5–6). D. OVX induced a 20% decrease in protein expression of neuronal glucose transporter (GLUT3), which is partially prevented by E2 treatment (50 µg/kg, SC). E. No change in expressions of glial glucose transporter (GLUT1_45 Kda_) between Sham-OVX, OVX and OVX+E2 groups.

Ovarian hormone loss is associated with cerebral metabolic decline [16], [17]. In the current study, we sought to determine whether loss of ovarian hormones and associated decline in brain metabolism were accompanied by a change in brain glucose uptake. To address this question, FDG-microPET imaging was conducted to determine the impact of ovarian hormone loss and E2 treatment (50 µg/kg, daily SC) on brain glucose uptake in 6-month 3×TgAD female mice. Compared to the Sham-OVX group, 5 weeks OVX induced a significant 25% decline in brain glucose uptake (Fig. 3A; 74.1±1.9% (OVX group) vs. 100±5.4% (Sham-OVX group), p<0.05, n = 7). Decline in brain glucose uptake was coincident with thermal dysregulation in the OVX condition (Fig. 2B). E2 treatment (50 µg/kg, daily SC) at the time of OVX partially prevented the OVX-induced decline in brain glucose uptake (Fig. 3A; 84.2±2.9% (E2 group) vs. 74.1±1.9% (OVX group), p<0.05, n = 4–7), consistent with the prevention of thermal dysregulation and weight gain (Fig. 2).

### Ovariectomy induced decrease in brain glucose transporter expression: prevention by 17β-estradiol

The OVX-induced decrease in brain glucose uptake could be attributed to multiple levels of metabolic dysregulation, including reduced glucose transport, compromised glycolysis, deficient mitochondrial capacity and impaired ATP generation [2], [13]–[15]. In these experiments, we determined the impact of ovarian hormone deprivation and E2 on the expression of glucose transporters in brain and neural cells via specific glucose transporters (GLUT) expressed in blood brain barrier (BBB) endothelial cells (GLUT1, 55 KDa), glial cells (GLUT1, 45 KDa) and neurons (GLUT3) [24].

To investigate whether the OVX-induced decline in brain glucose uptake is correlated with compromised glucose transport, expression of BBB GLUT1_55 Kda_, glial GLUT1_45 Kda_ and neuronal GLUT3 was assessed. BBB GLUT1_55 Kda_ expression was significantly decreased 38% by OVX (Fig 3B; 62.16±13.59% (OVX group) vs. 100±8.37% (Sham-OVX group), p<0.05, n = 5–6) in 3×TgAD female mice. Treatment with E2 completely prevented the OVX-induced decline in BBB GLUT1_55 Kda_ expression (Fig 3B; 96.42±5.56% (E2 group) vs. 62.16±13.59% (OVX group), p<0.05, n = 5–6). However, the expression level of GLUT1_55 Kda_ did not correlate with complete reversal of brain glucose uptake relative to Sham-OVX (Fig. 3A). This finding indicated that other key enzymes involved in brain glucose metabolism are required for complete reversal of the OVX-induced decline in glucose uptake.

OVX also induced a 20% decline (Fig 3C; 80.63±10.68% (OVX group) vs. 100±10.28% (Sham-OVX), p>0.1, n = 5–6) in neuronal GLUT3 expression, which was partially prevented by E2 (Fig 3C; 88.11±8.05% (E2 group) vs. 80.63±10.68% (OVX group), p>0.1, n = 5–6). Neither OVX nor E2 had a significant effect on the glial transporter GLUT1_45 Kda_ protein expression (Fig 3D). Collectively, these data indicate that ovarian hormone loss compromised glucose transport through the blood brain barrier (BBB) and neurons and spared glial glucose transporter expression. E2 prevented the OVX-induced decline of BBB GLUT1_55 Kda_ expression, with a partial preventative effect on neuronal GLUT3 expression. Further, the glial glucose transporter is not regulated by ovarian hormones as evidenced by no change in response to OVX and E2.

### Ovariectomy induced decrease in hexokinase expression and activity: prevention by 17β-estradiol

**Figure 4:**
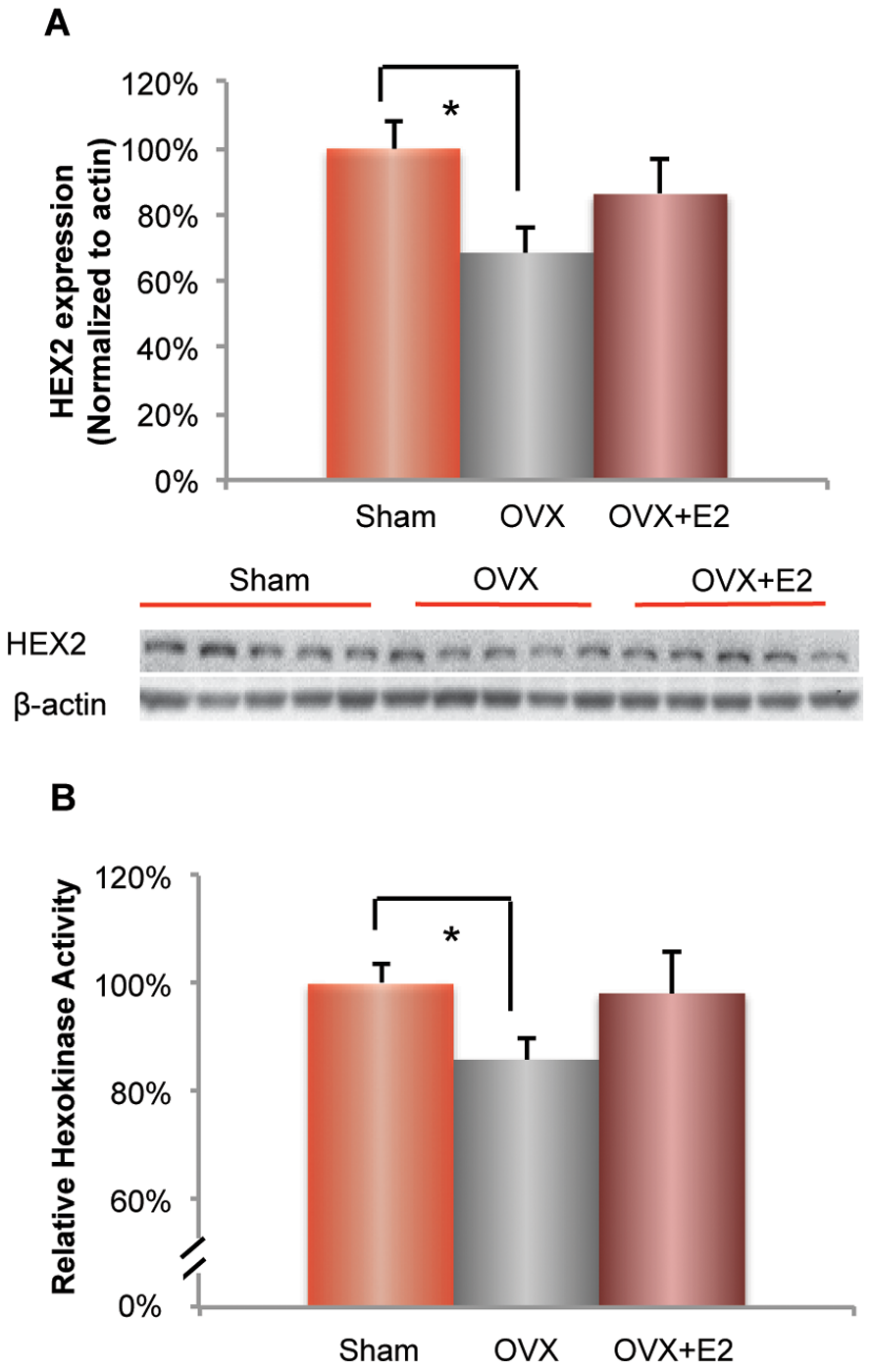
Ovariectomy (OVX) induced decrease in hexokinase expression and activity: prevention by 17β-estradiol. A. In female 3×TgAD brains, OVX induced a significant decrease in protein expression of hexokinase 2 (*, P<0.05, bars represent mean value±SEM, n = 5−6), which was partially prevented by E2 treatment. B. OVX induced a significant decrease in hexokinase activity (*, p<0.05, bars represent mean value ± SEM, n = 5–6), which was partially prevented by E2 treatment.

In neurons and glial cells, glucose is irreversibly phosphorylated to glucose-6-phosphate by hexokinase, which is the first and rate-limiting step in glycolysis. The glucose uptake signal in FDG-PET imaging is also dependent upon hexokinase activity, as cellular FDG accumulation is mediated by hexokinase that phosphorylates FDG to FDG-6-phosphate, which does not undergo further metabolism [25]. To investigate whether OVX-induced decline in glucose uptake is associated with compromised glucose phosphorylation, we first analyzed the expression of hexokinase type II, which is the isozyme sensitive to hormonal regulation [26]. In 3×TgAD female brain, OVX induced a significant 30% decline in hexokinase II protein expression (Fig 4A; 68.49±8.01% (OVX group) vs. 100±8.03% (Sham-OVX group), p<0.05, n = 5–6), which was partially but not significantly prevented by E2 (Fig 4A; 86.17±10.61% (E2 group) vs. 68.49±8.01% (OVX group), p = 0.11, n = 5–6). The OVX-induced decline in hexokinase expression was paralleled by a significant decline in hexokinase activity, although this 15% decline was not as great as the decline in protein expression (Fig 4B; 84.80±5.26% (OVX group) vs. 100±3.4% (Sham-OVX group), p<0.05, n = 5–6). As with protein expression, E2 partially but not significantly prevented the OVX-induced decline in hexokinase activity (Fig 4B; 96.07±7.43% (E2 group) vs. 84.80±5.26% (OVX group), p = 0.14, n = 5–6).

The magnitude of the OVX-induced decline in hexokinase protein expression and activity was positively correlated with the OVX-induced decline in brain glucose uptake (Fig. 3A). These data are consistent with the key role of hexokinase in the FDG-PET signal [27]. Further, the impact of E2 on preventing decline in HEX protein expression and activity is consistent its partial prevention of the OVX-induced decline in FDG-PET signal.

### Ovariectomy induced a shift in LDH1 and LDH5 ratio: prevention by 17β-estradiol

**Figure 5:**
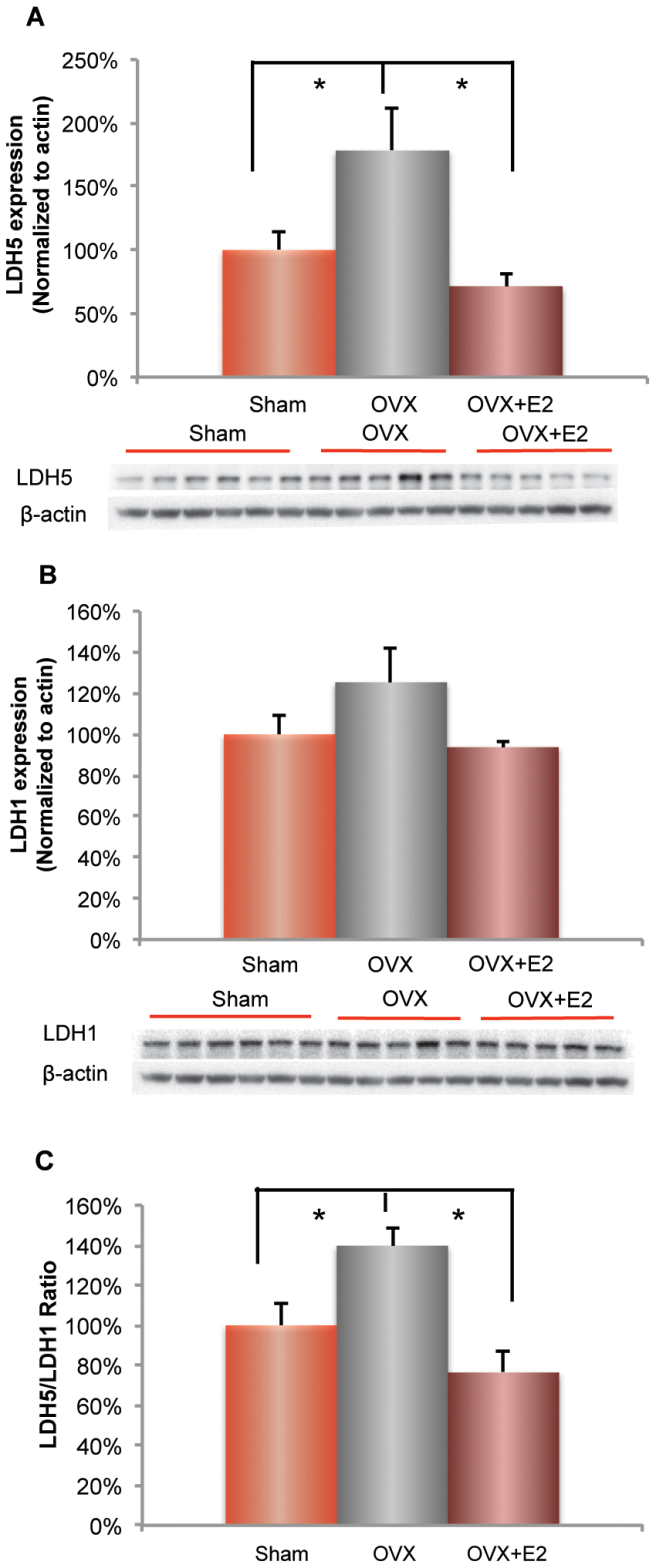
Ovariectomy (OVX) induced a shift in LDH1 and LDH5 ratio and prevention by 17β-estradiol. A. OVX induced a significant increase (*, p<0.05, bars represent mean value ± SEM, n = 5–6) in the expression of glial lactate dehydrogenase 5 (LDH5) expression. B. OVX induced a 25% increase in neuronal lactate dehydrogenase 1 (LDH1). C. The lactate production indicator, LDH5/LDH1 ratio increased significantly (*, p<0.05, bars represent mean value ± SEM, n = 5–6). All OVX-induced changes were prevented by E2 treatment.

Decreased glucose supply and utilization could activate alternative metabolic pathways to compensate for decline in brain glucose metabolism. As we and others have shown, neurons can utilize lactate or ketone bodies as alternative fuels [19], [28]–[30]. In response to energetic demand, brain can utilize lactate to sustain synaptic transmission, whereas under prolonged glucose deprivation, the brain will utilize ketone bodies generated from the liver to support the energetic demand [31]. To investigate whether OVX induced a shift in utilization of alternative fuels, we first determined lactate dehydrogenase protein expression level in the Sham-OVX, OVX and OVX+E2 mice. Multiple isoforms of lactate dehydrogenase are expressed in glial and neuronal cell types, which enable these cells to generate and utilize lactate. The LDH5 isoform, which is composed of four A subunits, converts pyruvate to lactate in glial cells. LDH1 isoform, which is formed by four B subunits, converts lactate to pyruvate for ATP generation in neurons [32], [33]. In the OVX condition, LDH5 glial cell expression was dramatically increased (Fig 5A; 178±33.34% (OVX group) vs. 100±14.51% (Sham-OVX group), p<0.05, n = 5–6). In neurons, LDH1 expression exhibited a similar increase in expression, although not significant (Fig 5B; 125.22±16.32% (OVX group) vs. 100±8.69% (Sham-OVX group), p = 0.09, n = 5–6).

An increased LDH5/LDH1 ratio can be indicative of elevated lactate in the mouse and human brain, and is consistent with an aging phenotype [34], [35]. Based on the elevated expression of LDH5 to LDH1, we tested whether there was a significant increase in LDH5 to LDH1 ratio. The ratio of LDH5/LDH1 expression was significantly increased in the OVX condition relative to Sham-OVX (Fig 5C; 139.70±9.37% (OVX group) vs.100±10.72% (Sham-OVX group), p<0.05, n = 5–6). Animals treated with E2 at the time of OVX did not exhibit an increase in the LDH5/LDH1 ratio and were indistinguishable from Sham-OVX (Fig 5C; 76.36±10.90% (E2 group) vs.100±10.72% (Sham-OVX group), p = 0.16, n = 5–6). Under OVX condition, the increased expression of the enzyme LDH5 required for conversion of pyruvate to lactate by glial cells is predicative of greater lactate generation relative to consumption in neurons, suggesting the potential of lactate accumulation in the hippocampus. Prevention of the rise in LDH5 expression and prevention of the increase in LDH5/LDH1 ratio by E2 is predicative of balanced lactate generation to utilization in hippocampus.

### Ovariectomy activated the ketogenic pathway: prevention by 17β-estradiol

**Figure 6:**
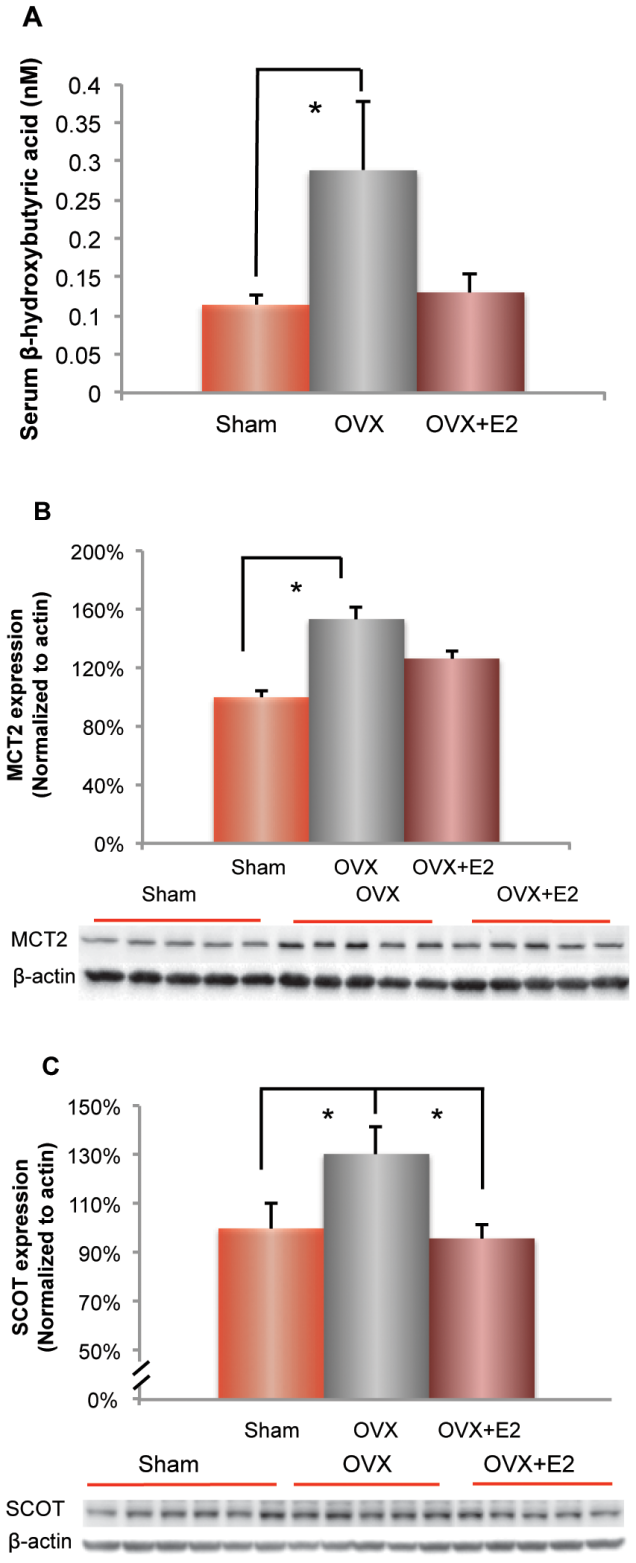
Ovariectomy (OVX) induced activation of ketogenic pathway and prevention by 17β-estradiol. A. OVX induced a significant increase (*, p<0.05, bars represent mean value±SEM, n = 5–6) in serum β-hydroxybutyrate level, which was prevented by E2 treatment. B. OVX induced a significant increase (*, p<0.05, bars represent mean value ± SEM, n = 5–6) in the expression of neuronal monocarboxylate transporter 2 (MCT2), which was prevented by E2 treatment. C. OVX induced a significant increase (*, p<0.05, bars represent mean value ± SEM, n = 5–6) in the expressions of 3-oxoacid-CoA transferase (SCOT), which was prevented by E2 treatment.

Under prolonged glucose deprivation, the brain can utilize ketone bodies as energy substrates. To determine whether OVX induced a shift in utilization to ketone bodies, we determined ketone body level and the expression of the ketone body transporter MCT2 and the metabolic enzyme Succinyl-CoA: 3-ketoacid CoA transferase (SCOT) required to convert ketone bodies to acetyl-CoA. To assess liver generation of ketone bodies as an alternative fuel for utilization by brain, we determined the level of β-hydroxybutyrate in the plasma [36]. OVX induced a significant 150% rise in the serum level of β-hydroxybutyrate (Fig 6A; 0.2886±0.0897 (OVX group) vs. 0.1142±0.0132 (Sham-OVX group), p<0.05, n = 5–6), the major circulating ketone body generated by the liver. In parallel with the increased ketone levels, protein expression of neuronal monocarboxylate transporter (MCT2), which transports ketone bodies or lactate, was increased by 50% in the OVX condition (Fig 6B; 153.34±7.63% (OVX group) vs. 100±4.41% (Sham-OVX group), p<0.05, n = 5–6). SCOT, the key enzyme that catabolizes ketone bodies to acetyl-CoA for subsequent ATP generation, was also increased by OVX (Fig 6C; 130.40±11.60% (OVX group) vs. 100±10.60% (Sham-OVX group), p = 0.09, n = 5–6). Treatment with E2 initiated at the time of OVX prevented OVX-induced increase in β-hydroxybutyrate level (Fig 6A; 0.1304±0.0232 (E2 group) vs. 0.2886±0.0897 (OVX group), p = 0.06, n = 5–6) and expression of MCT2 (Fig 6B; 126.42±9.48% (E2 group) vs. 153.34±7.63% (OVX group), p = 0.06, n = 5). E2 treatment also significantly prevented the protein expression of SCOT (Fig 6C; 95.84±5.54% (E2 group) vs. 125.14±14.25% (OVX group), p<0.05, n = 5–6). Collectively, the data indicate that OVX induces a significant rise in the generation of ketone bodies, the enzyme required to convert ketone bodies to acetyl-CoA and transport of ketone bodies into neurons. E2 treatment at the time of OVX prevented the rise in the ketogenic system.

### Ovariectomy induced increase in β-amyloid levels in brain: prevention by 17β-estradiol

**Figure 7:**
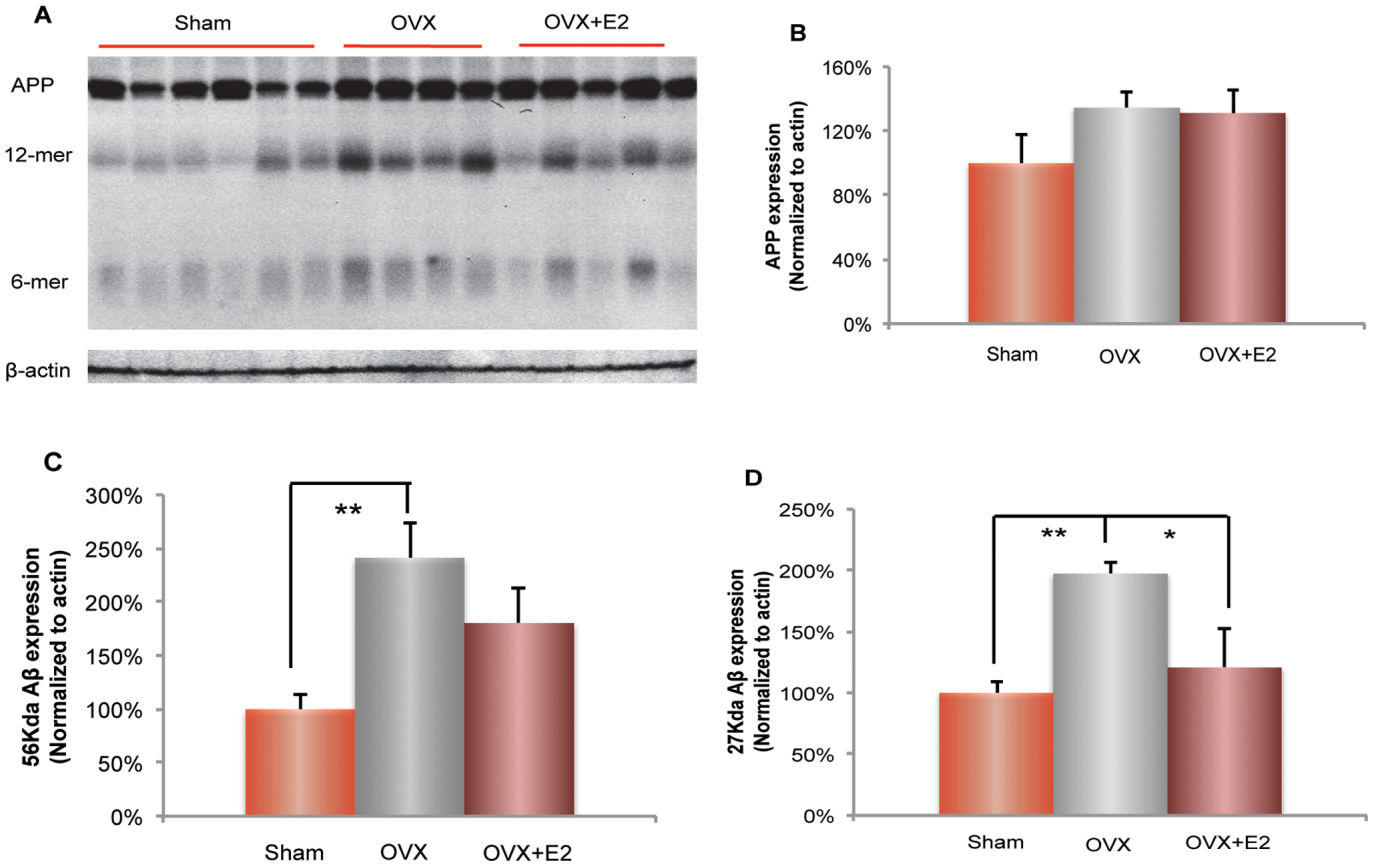
Ovariectomy (OVX) induced increase in β-amyloid level: prevention by 17β-estradiol. A. Three Aβ related bands were characterized: full length APP band (∼100 Kda), Aβ dodecamers (56 Kda) and Aβ hexamers (27 Kda). B. There is no significant change of APP expressions between Sham-OVX, OVX and OVX+E2 groups. C. OVX induced a significant increase (**, p<0.01, bars represent mean value ± SEM, n = 4–6) in the expression of 56 Kda Aβ. D. OVX induced a significant increase (**, p<0.01, bars represent mean value ± SEM, n = 4–6) in the expression of 27 Kda Aβ, which was partially prevented by E2 treatment (*, p<0.05; bars represent mean value±SEM, n = 4–5).

Previous analyses demonstrated that female 3×TgAD mouse model showed an age-related increase in β-amyloid deposition in brain [1]. To investigate whether an increase in β-amyloid level is associated with ovarian hormone loss, we assessed the β-amyloid oligomers in the 3×TgAD cortex samples. We characterized three major Aβ related bands: full length APP band (∼100 Kda), Aβ dodecamers (56 Kda) and Aβ hexamers (27 Kda) (Fig. 7A). There was no significant difference in APP expression across all three groups (Fig. 7B). Compared to the Sham OVX, OVX induced a significant increase in the protein level of Aβ oligomers (Fig. 7C and 7D; 56 Kda Aβ: 241±32.3% (OVX group) vs. 100±13.3% (Sham-OVX group), p<0.01; 27 Kda Aβ: 197±8.7% (OVX group) vs. 100±8.7% (Sham-OVX group), p<0.01; n = 4–6). E2 treatment partially prevented the OVX-induced increase in Aβ oligomer levels (Fig. 7C and 7D; 56 Kda Aβ: 180±33.7% (E2 group) vs. 241±32.3% (OVX group), p = 0.11; 27 Kda Aβ: 120±32.3% (E2 group) vs. 100±8.7% (OVX group), p<0.05; n = 4–5). Data of analyses suggest that changes in ovarian hormone status, such as OVX and E2 treatment, induced alteration in the amyloidogenic pathway and/or Aβ clearance pathway, rather than directly regulating APP availability.

### 17β-estradiol upregulated bioenergetic gene expression: a broader impact on brain energy production

**Figure 8:**
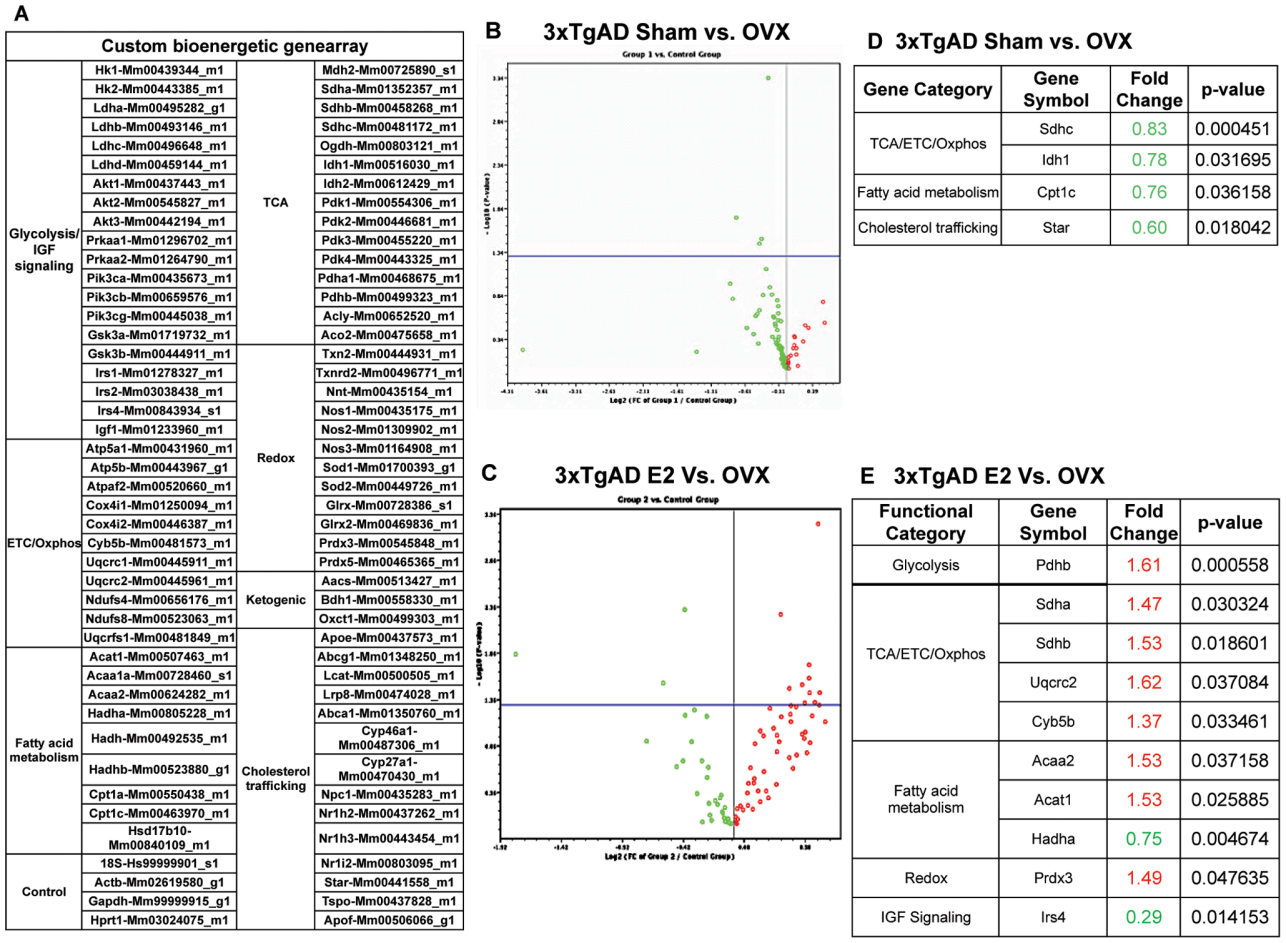
E2 treatment significantly regulated Bioenergetic gene expression. A. Gene list of custom bioenergetic genearray. B. Gene expression data presented in the valcano plot which displays fold changes vs p-values between Sham-OVX and OVX demonstrated that OVX had little impact on the expression of the bioenergetic genes. C. Gene expression data presented in the valcano plot which displays fold change vs p-values between OVX+E2 and OVX demonstrated that E2 treatment exerted a broad impact on the expression of genes involved in bioenergetic pathways. Each dot representes one individual gene; Red: up-regulated gene expression compared to OVX; Green: down-regulated gene expression compared to OVX; Dots above the bule line represent genes that are significantly regulated. D. Compared to OVX group, genes that were significantly changed in Sham-OVX group were categorized into 3 different functional groups: TCA/ETC/Oxphos (tricarboxylic acid cycle/electron transport chain/oxidative phosphorylation), fatty acid metabolism, and cholesterol trafficking. Data is presented as fold change relative to the OVX group with the corresponding p value listed for each individual gene. E. Compared to OVX group, genes that were significantly changed in OVX+E2 group were categorized into 5 different functional groups: glycolysis, TCA/ETC/Oxphos, fatty acid metabolism, redox and IGF signaling. Data is presented as fold change relative to the OVX group with the corresponding p value listed for each individual gene.

To investigate at a systems level the impact of ovarian hormone loss and E2 treatment on the bioenergetic profile in 3×TgAD mouse brains, we designed a custom bioenergetic low-density gene array and conducted real time RT-PCR analyses to assess the expression of key genes involved in brain bioenergetics (Fig. 8A). Gene expression data presented in the volcano plot displays fold change vs. p-value (Fig. 8B and Fig. 8C), with significantly changed genes listed respectively (Fig. 8D and Fig. 8E). Results presented in the volcano plot demonstrated that when compared to Sham-OVX, OVX had a relatively small impact with 4 genes (Sdhc, Idh1, Cpt1c, Star) that exhibited significant changes (Fig. 8B and Fig. 8D). E2 treatment induced a much broader response with a total of 10 genes significantly changed when compared to the OVX group, including 1 gene involved in glycolysis (Pdhb), 4 genes involved in TCA and electron transport chain (ETC) (Sdha, Sdhb, Uqcrc2, Cyb5b), 3 genes involved in fatty acid metabolism (Acaa2, Acat1, Hadha), and 2 genes involved in cellular redox and insulin signaling (Prdx3 and Irs4 respectively) (Fig. 8C and Fig. 8E).

## Discussion

**Figure 9:**
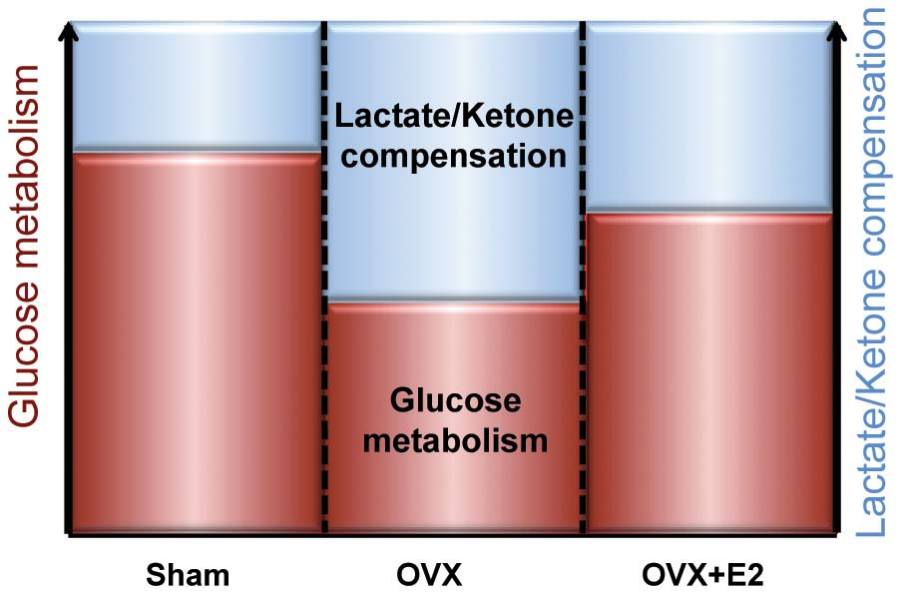
Impact of ovariectomy on bioenergetic system of 3×TgAD female brain. Ovariectomy (OVX) induced a decline in brain glucose metabolism as shown by the functional microPET imaging and biochemical analyses. This decline is paralleled by an activation of the lactate/ketone compensatory pathway. E2 treatment partially prevented the OVX-induced decline in brain glucose metabolism and partially prevented the activation of ketogenic pathway.

In this study, we demonstrated that loss of ovarian hormones induced a decline in glucose-driven brain bioenergetics, which was associated with dysregulated body weight and temperature control. We further identified a compensatory shift from glucose-driven brain bioenergetics towards an alternative substrate pathway (lactate and ketone bodies) induced by ovarian hormone loss. Although E2 treatment completely prevented the dysregulation of body weight and temperature control, the efficacy of E2 to prevent decline in on bioenergetic capacity of the brain was partial (Fig. 9).

Classic indicators of ovarian hormone loss occurred in the ovariectomized 3×TgAD mice, evidenced by an increase in body weight and tail skin temperature within 5 weeks, a time frame comparable to 2.5 years of human life [37], [38]. These results are consistent with clinical observations of the metabolic and thermal dysregulation in surgically oophorectomized premenopausal women [39], [40].

It is well known that the decline in brain glucose metabolism is associated with decreased cognitive function in mild cognitive impairment and Alzheimer's disease [3]–[7], [41]–[43] and, at least, partially contributes to the neurodegenerative process [31]. ApoE ε4 carriers, who are cognitively normal but at increased risk for AD, exhibited deficits in cerebral metabolic rate for glucose (CMRglu) in posterior cingulate (PCC) and parietal temporal cortex several decades prior to onset of dementia [7]. Further, cognitively normal people with a maternal family history AD, who have a higher risk for AD [44], exhibited glucose hypometabolism in brain regions identified in prodromal AD [41], [45]. Collectively, these data indicate that hypometabolism in brain is an early and persistent indicator of both risk for and severity of Alzheimer's disease [3]–[7], [41].

Results of our analyses to determine the impact of OVX on expression of glucose transporters in blood brain barrier, neurons and glial cells indicated a significant reduction in blood brain barrier specific glucose transporters. The 55 Kda form of GLUT1 mediates glucose transport across the blood brain barrier (BBB) and is a rate-limiting step for brain glucose utilization under metabolic demand [46], [47]. Findings from the current study in a mouse model of familial AD are consistent with a decline in BBB GLUT1_55 Kda_ expression in late stage AD and compromised BBB glucose transport [48]. In patients with GLUT1 deficiency syndrome, GLUT1 haplo-insufficiency leads to a global decrease in cortical glucose uptake and multiple neurological deficits [49], [50]. In addition to GLUT1, GLUT3 is also associated with the pathogenesis of Alzheimer's [51]. In the current report, 5-week OVX induced a significant (38%) decrease in the BBB GLUT1_55 Kda_ protein, whereas E2 treatment initiated at the time of OVX prevented the OVX-induced decline. In contrast to the BBB GLUT1_55 Kda_, the neuronal glucose transporter GLUT3 and the glial glucose transporter GLUT1_45 Kda_ were not significantly affected by 5-week OVX or E2 treatment. The decline in BBB GLUT1_55 Kda_ in the face of sustained glial and neuronal GLUT expression is indicative of compromised BBB glucose transport as an early response to ovarian hormone loss. These results indicate a key role of BBB GLUT1_55 Kda_ in mediating the decline in the FDG-PET detected brain glucose uptake induced by ovarian hormone loss.

In addition to glucose transporters, cerebral glucose utilization is under tight control by hexokinase, which serves as the first rate-limiting step in glycolysis. In the current study, ovarian hormone deprivation induced a significant decline in hexokinase protein expression as well as activity, which was prevented by E2 treatment in 3×TgAD female mice. Hexokinase interaction with the mitochondrial VDAC also plays an important role in preventing mitochondria-mediated apoptosis and promoting cell survival in neurons and other cell types [52]–[54]. AD patients exhibit declined hexokinase activity in the brain, cerebral microvessel, leukocytes and fibroblasts [55]–[57]. Previous studies have demonstrated that ovarian hormone loss led to compromised-brain hexokinase activity, whereas short-term estrogen treatment was efficacious to increase hexokinase activity in rat brains [12]. Collectively, these data demonstrate that the reduction in BBB GLUT1_55 Kda_ expression and hexokinase activity are key drivers for the decline in brain glucose uptake.

Lactate is a well described bioenergetic fuel supplied by glia to neurons and is particularly important under conditions of high synaptic activity, such as occurs during learning and memory [58]. Further, lactate can serve as an auxiliary fuel during glucose insufficiency by metabolism of glycogen stores to generate glucose and subsequently lactate [59]. In the current study, we found that OVX induced an 80% increase in LDH5, suggestive of increased production of lactate in glial cells for use as a compensatory substrate in neurons. However, LDH1 expression, presumably neuronal, was modestly although not significantly increased following OVX. These findings suggest that glial cells are likely to produce lactate in excess to its utilization by neurons. Increased LDH5 expression relative to LDH1 is consistent with an accelerated aging phenotype [34]. This postulate is supported by data derived from a premature aging model of mitochondrial mutation with high levels of mtDNA point mutations and linear deletions. In this model, increased LDH5/LDH1 ratio was associated with high levels of lactate in brain, which paralleled that observed in aging [34]. In the current study, the increased LDH5/LDH1 ratio in the OVX condition suggests that ovarian hormone loss leads to lactate accumulation and an accelerated-aging phenotype in the 3×TgAD female brain.

Under conditions of prolonged glucose deprivation, the brain shifts to utilization of ketone bodies as an alternative fuel. Previous findings from our group demonstrated that the bioenergetic fuel shift occurred in the 3×TgAD brain from the outset and represents an AD metabolic phenotype [19]. In the current report, we observed an increase in serum ketone body concentration, which was accompanied by a simultaneous increase in hippocampal expression of SCOT, the rate-limiting enzyme for conversion of ketone bodies to acetyl-CoA for entry into the TCA cycle. In addition, the neuronal MCT2 transporter for ketone bodies and lactate was also increased. These changes indicate a coordinated upregulation of peripheral ketone supply, cellular transport mechanism and conversion of ketone bodies to compensate for decline in glucose metabolism in brain. E2 treatment prevented this shift to the ketogenic pathway in brain, likely via preventing decline of the aerobic glycolytic pathway. Collectively, these data demonstrated that ovarian hormone loss induced an accelerated AD phenotype, which was partially prevented by E2 treatment.

β-amyloid deposition is the pathological marker of AD and is proposed to be responsible for cognitive deficits in AD [60]–[62]. Consistent with the previous findings in hippocampus, the current study demonstrated that OVX activates amyloidogenic pathway in cortex, indicating that ovarian hormone loss and E2 treatment induced a brain-wide systems response in the amyloidogenic pathway as well as the Aβ clearance pathway [2]. In addition, increased β-amyloid levels paralleled the decline in hexokinase activity. Previous studies reported that β-amyloid triggers the release of neuronal hexokinase-1 from mitochondria and inactivates hexokinase-1 [62]. Taken together, these results provide a plausible mechanism underlying clinical observations that decline in brain glucose metabolism is associated with an increase in β-amyloid deposition [30], [63].

Gene expression analyses demonstrated that genes involved in the TCA cycle were increased in the OVX group (Figure 8D), suggesting a potential compensatory activation of acetyl-CoA utilization in response to decreased glucose transport and compromised glycolysis induced by OVX. E2 treatment significantly increased the expression of genes in Complex II (Sdha and Sdhb) and Complex III (Uqcrc2) (Figure 8E), suggesting an up-regulation in ETC function. Together with the observation that E2 treatment prevented decline in glucose transporters and hexokinase activity in cytosolic compartment, these data suggest a system-level regulation of brain glucose metabolism and bioenergetic function by E2 in multiple function domains spanning from glucose transport to glycolysis and to ETC.

In the current study, E2 treatment in the 3×TgAD brain partially prevented OVX-induced decline in whole brain glucose uptake and a shift to alternative substrates in the hippocampus, whereas E2 treatment completely prevented dysregulation in temperature and body weight. The E2 prevention of dysregulation of temperature and body weight indicates that the E2 dose and regimen was appropriate to regulate hypothalamic functions. The dissociation between hypothalamic and hippocampal response to E2 could be explained by: 1) a different E2 dose requirement; 2) the 3×TgAD female brain has a diminished response to E2; 3) the necessity for another ovarian hormone, progesterone, as was shown for prevention of OVX-induced rise in β-amyloid [64].

The purpose of the current study was to determine whether bioenergetic deficits in mitochondrial function previously observed [2] were accompanied by changes in glucose availability to sustain aerobic glycolysis and whether there was activation of compensatory responses for the transport and metabolism of alternative fuels. Collectively, the data demonstrate that loss of ovarian hormones led to a significant decline of glucose uptake, glucose transporter expression and in the enzymes required for glucose metabolism. Coincident with the decline in glucose uptake, transport and metabolism induced by OVX, there was a significant rise in the transporter for alternative fuels and the enzymes required for their metabolism. These results together with our previous findings demonstrate that loss of ovarian hormones is associated with a system wide decline in glucose transport and utilization in brain and a compensatory rise in the systems required to utilize alternative, less efficient fuels and induced an accelerated AD phenotype.
